# Genome-Wide Analysis of Left Ventricular Maximum Wall Thickness in the UK Biobank Cohort Reveals a Shared Genetic Background With Hypertrophic Cardiomyopathy

**DOI:** 10.1161/CIRCGEN.122.003716

**Published:** 2023-01-04

**Authors:** Nay Aung, Luis R. Lopes, Stefan van Duijvenboden, Andrew R. Harper, Anuj Goel, Christopher Grace, Carolyn Y. Ho, William S. Weintraub, Christopher M. Kramer, Stefan Neubauer, Hugh C. Watkins, Steffen E. Petersen, Patricia B. Munroe

**Affiliations:** William Harvey Research Institute, Barts and The London School of Medicine and Dentistry (N.A., S.v.D., S.E.P., P.B.M.).; National Institute for Health and Care Research, Barts Cardiovascular Biomedical Research Centre, Queen Mary University of London (N.A., S.v.D., S.E.P., P.B.M.).; Barts Heart Centre, St Bartholomew’s Hospital, Barts Health NHS Trust, West Smithfield (N.A., L.R.L., S.E.P.).; Centre for Heart Muscle Disease, Institute of Cardiovascular Science, University College London (L.R.L.).; Radcliffe Department of Medicine, Division of Cardiovascular Medicine (A.R.H., A.G., C.G., S.N., H.C.W.).; Wellcome Centre for Human Genetics, University of Oxford, United Kingdom (A.R.H., A.G., C.G., H.C.W.).; Cardiovascular Division, Department of Medicine and Department of Radiology, Brigham and Women’s Hospital, Boston, MA (C.Y.H.).; MedStar Heart and Vascular Institute, Washington, DC (W.S.W.).; Cardiovascular Division, University of Virginia Health System, Charlottesville (C.M.K.).; NIHR Oxford Biomedical Research Centre, Oxford University Hospitals NHS Foundation Trust, John Radcliffe Hospital, United Kingdom (S.N., H.C.W.).

**Keywords:** cardiovascular magnetic resonance, hypertrophic cardiomyopathy, odds ratio, loci, risk score

## Abstract

Left ventricular maximum wall thickness (LVMWT) is an important biomarker of left ventricular hypertrophy and provides diagnostic and prognostic information in hypertrophic cardiomyopathy (HCM). Limited information is available on the genetic determinants of LVMWT.

**Methods:**

We performed a genome-wide association study of LVMWT measured from the cardiovascular magnetic resonance examinations of 42 176 European individuals. We evaluated the genetic relationship between LVMWT and HCM by performing pairwise analysis using the data from the Hypertrophic Cardiomyopathy Registry in which the controls were randomly selected from UK Biobank individuals not included in the cardiovascular magnetic resonance sub-study.

**Results:**

Twenty-one genetic loci were discovered at *P*<5×10^−8^. Several novel candidate genes were identified including *PROX1*, *PXN*, and *PTK2*, with known functional roles in myocardial growth and sarcomere organization. The LVMWT genetic risk score is predictive of HCM in the Hypertrophic Cardiomyopathy Registry (odds ratio per SD: 1.18 [95% CI, 1.13–1.23]) with pairwise analyses demonstrating a moderate genetic correlation (r_g_=0.53) and substantial loci overlap (19/21).

**Conclusions:**

Our findings provide novel insights into the genetic underpinning of LVMWT and highlight its shared genetic background with HCM, supporting future endeavours to elucidate the genetic etiology of HCM.

Left ventricular hypertrophy (LVH), either defined by increased mass or increased wall thickness due to cardiomyocyte hypertrophy, is a key myocardial remodeling process in both normal physiological adaptation and pathological states such as hypertensive heart disease, aortic stenosis, and cardiomyopathies.^1^ LVH is a well-established marker of adverse prognosis including heart failure and arrhythmia.^1,2^ It has significant heritability, estimated at 20% to 40% for left ventricular (LV) mass derived from imaging and higher with ECG-based measurements.^3^ To elucidate relevant molecular pathways, a limited number of genome-wide association studies (GWASs) have been performed for LVH. Different measures of LVH have been used, including crude endophenotypes based on ECG criteria^4–6^ and echocardiographic estimates of ventricular mass.^7–11^ Studies using echocardiographic measurements of LV mass or LV wall thickness have discovered a small number of loci but none was replicated in an independent sample or in a combined meta-analysis.^12–14^ In contrast, a large genome-wide association meta-analysis of multiple ECG-based LVH traits reported 52 loci.^4^ These prior studies were hampered by suboptimal phenotypic precision due to the geometric assumptions in two-dimension echocardiography^15^ or poor sensitivity of ECG-LVH criteria.^16^

Cardiovascular magnetic resonance (CMR) is the gold-standard imaging modality for the measurement of cardiac volumes and function and allows for optimal myocardial-blood (endocardial and epicardial borders) definition. Recent machine learning techniques allow rapid analysis of a large quantity of CMR imaging data with an accuracy comparable to human experts.^17,18^ This creates enhanced opportunities to conduct large-scale population imaging studies to unravel the genetic basis of cardiac phenotypes. Leveraging the automated analysis of CMR studies, we have previously identified one locus (rs2255167, *TTN*) for LV mass and three loci for LV mass to volume ratio, a remodeling phenotype, in a sample of >16 000 individuals in the UK Biobank^19^; LV maximum wall thickness (LVMWT) was not analyzed in this study.

Hypertrophic cardiomyopathy (HCM) is a relatively common genetic heart muscle disease and often caused by rare pathogenic variants in sarcomere genes (sarcomere-positive HCM), although many patients do not have pathogenic variants identified by genetic testing (sarcomere-negative HCM). HCM is a significant cause of sudden cardiac death and heart failure. Increased LVMWT (≥15 or ≥13 mm in relatives) not only defines the diagnosis but also reflects phenotypic severity and is used for estimating sudden cardiac death risk.^20^ In the UK Biobank population cohort, the prevalence of LVMWT ≥15 mm is approximately 0.1%.^21^ Two recent HCM GWASs^22,23^ described the influence of common variations on HCM phenotype. One study^22^ found 12 genome-wide significant loci in a case control analysis of 2780 cases and 47 486 controls and reported a significant polygenic effect in sarcomere-negative HCM. The other study^23^ found 16 loci associated with HCM from both a single-trait analysis in 1733 cases and 6628 controls and joint multi-trait analyses combining nine LV imaging traits including LV mean wall thickness in 19 260 individuals. Neither of these studies systematically investigated the genetic basis of LVMWT as the main phenotype.

In this study, we conducted a GWAS to identify genetic susceptibility loci for CMR-derived LVMWT (Figure 1) in a general population (UK Biobank).^24^ We next evaluated the relationships of identified loci with HCM in a well-defined HCM population from the Hypertrophic Cardiomyopathy Registry.^25^

**Figure 1.**
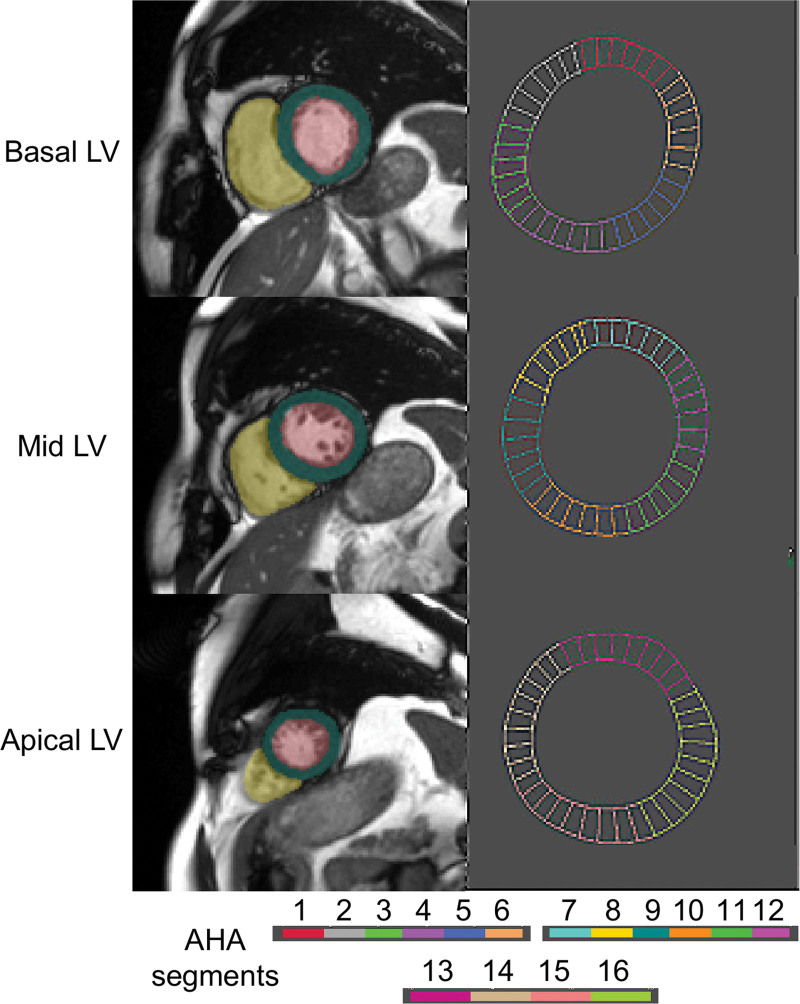
**Automatic segmentation and derivation of left ventricular maximum wall thickness (LVMWT) in AHA-16 segment model.** Images in the left-hand side show short-axis steady-state free precession cine cardiovascular magnetic resonance images of left and right ventricles with automatic segmentation masks (green=left ventricular myocardium, red=left ventricular blood pool and yellow=right ventricular blood pool) in the basal, mid and apical slices. Images in the right-hand side illustrate the corresponding left ventricular (LV) wall thickness measurements in the AHA-16 segment model.

## Methods

GWAS summary statistics will be deposited in GWAS Catalog (https://www.ebi.ac.uk/gwas/). The analytic methods and study materials generated in this study were part of UK Biobank application number 2964 and will be returned to UK Biobank where it can be accessed per request. Please see the UK Biobank’s website for the detailed access procedure (http://www.ukbiobank.ac.uk/register-apply/). The study complies with the Declaration of Helsinki and was approved by our institutional review body. All participants provided informed written consent. Detailed methods are available in the Supplemental Methods.

## Results

The clinical characteristics of UK Biobank cohort are presented in Table S1. A total of 42 176 European individuals (mean age 64 years, 48% men) with CMR studies were included in the discovery GWAS. One-third of the study sample had a known history of systemic hypertension. The mean (SD) of LVMWT is 9 (2) mm. Only 2% of the cohort has LVMWT ≥13 mm.

### Genomic Loci Associated With LVMWT

**Figure 2.**
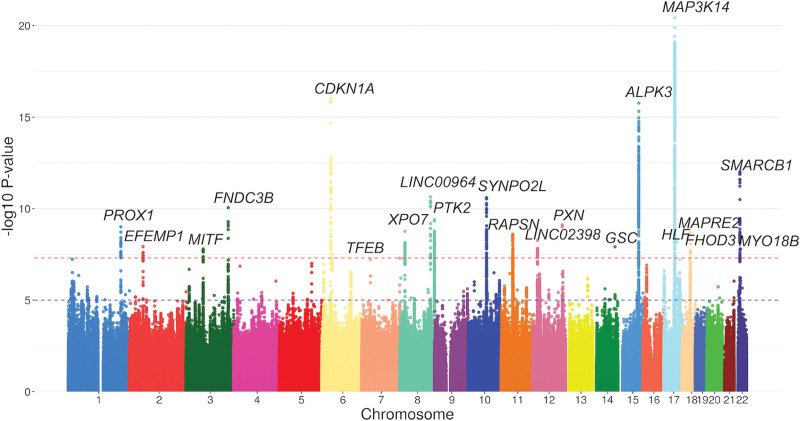
**Manhattan plot of left ventricular maximum wall thickness genomic loci.** Each point represents a genetic variant. The red line indicates the genome-wide significant threshold at *P*<5×10^−8^ and the blue line indicates the suggestive significance at *P*<1×10^−5^. *P* values are 2-sided based on the Chi-square test statistics in the BOLT-LMM software.

The estimated narrow-sense heritability ($h_{g}^{2}$ SNP [SD]) of LVMWT was 0.26 (0.01). The GWAS discovered 21 loci at *P*<5×10^−8^ (Table 1; Figure 2). We observed no evidence of population stratification or cryptic relatedness (genomic inflation factor [λ]=1.097, linkage disequilibrium score regression intercept=1.02, quantile-quantile plot in Figure S1). Although there appeared to be approximately 10% inflation of association statistics (λ 1.097), the linkage disequilibrium score regression intercerpt is low at 1.02, indicating that the test statistic inflation was likely caused by polygenicity rather than confounding biases. The quantile-quantile plot shape also improved to an expected pattern after removing a locus (*MAP3K14*) with a dense linkage disequilibrium (LD) block (Figure S1). Conditional analyses to identify secondary independent variants revealed one secondary variant each at two loci (*LINC02398* and *MAP3K14*; Table S2, resulting in a total of 23 independent variants in 21 loci (the LocusZoom^26^ plots in Figure S2). The trait variance explained by the lead and secondary variants was 1.1%.

**Table 1.**
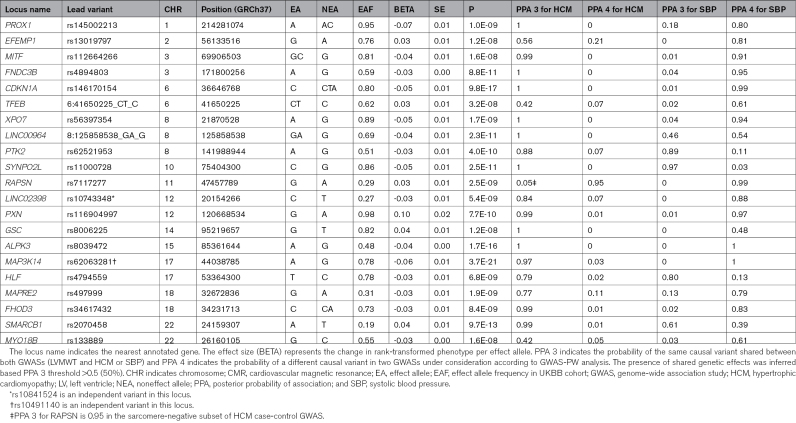
Genomic Loci Associated With CMR-Derived LV Maximum Wall Thickness

### Functional Annotation of Variants

The LVMWT loci contained a total of 4048 variants in the 99% credible sets: 1.4% exonic variants, 81% intronic or regulatory region variants, 1.7% intergenic variants and the remainder were upstream and downstream gene variants, 5’ and 3’ UTR variants (Table S3). Among 56 exonic variants, only one missense variant (rs3812629 in *SYNPO2L* locus) was predicted to be damaging by both SIFT and PolyPhen-2 bioinformatic tools. Out of 3,992 non-coding variants, 1172 variants were considered functionally important by CADD or RegulomeDB; however, most of these variants (80%) belonged to *MAP3K14* locus due to its dense LD structure. The eQTL colocalization analysis, which leveraged gene expression data in arterial, left ventricular and left atrial appendage tissues, detected one variant each for *GSC* and *SMARCB1* (rs8006225 and rs2070458, respectively) and two variants in high LD (r^2^>0.9) for *LINC00964* locus (rs34866937 and rs35006907).

### Genetic Overlap With Other Traits

We first interrogated the Phenoscanner and GWAS Catalog databases to examine the pleiotropic associations between our lead variants and their close proxies (LD r^2^≥0.8) and other traits. Here, we highlight a few pertinent associations. Three LVMWT loci (*RAPSN*, *LINC02398* and *SYNPO2L*) harbored variants associated with systemic hypertension or blood pressure measurements according to the Phenoscanner database (Table S4). In the GWAS Catalog, variants in seven loci (*MITF*, *FNDC3B*, *CDKN1A*, *SYNPO2L*, *MAP3K14*, *FHOD3* and *SMARCB1*) were associated with HCM. Variants in *LINC00964*, *PXN*, *ALPK3*, *MAP3K14* and *SMARCB1* loci showed associations with other LV imaging parameters including LV mass, LV volume and LV ejection fraction (Table S5). The overlap between known genetic associations reported in the Phenoscanner and the GWAS Catalog and our results was illustrated in Figure S3. We also performed a lookup of our genome-wide significant variants and proxies in a recently published GWAS of LV mean wall thickness,^23^ which showed an overlap of four loci (*ALPK3*, *MAP3K14*, *SMARCB1*, *MYO18B*) at a genome-wide significance level (*P*<5×10^−8^) and a further three loci (*MITF*, *CDKN1A*, *PXN*) at a suggestive *P*<1×10^−5^ (Table S6). Three loci (*PROX1*, *GSC*, *HLF*) can be considered completely novel based on the absence of known associations with other traits.

### Candidate Gene Identification and Pathway Enrichment

Candidate gene mapping at GWAS loci using a diverse set of bioinformatic evidence identified a total of 80 protein encoding genes. Further details on these candidate genes were outlined in Table S7. Three genes (*SYNPO2L*, *MAPT*, *KANSL1*) were prioritised by the presence of damaging non-synonymous variants; three protein-coding genes (*MTSS1*, *GSC*, *MMP11*) were highlighted by the eQTL colocalization analysis. Six genes (*MITF*, *FNDC3B*, *PTK2*, *RAPSN*, *ALPK3* and *MYO18B*) were supported by more than two different sources of bioinformatic evidence. The Data Driven Expression Prioritised Integration for Complex Traits pathway analysis including all GWAS variants at *P*<1×10^−5^ highlighted the enrichment of genes related to pericardial effusion at FDR<0.01 (Table S8). Functional profiling of all candidate genes in g:Profiler revealed enrichment in the biological pathways associated with sarcomere organization and muscle development (Table S9).

### Phenome-Wide Association Study

In our locus-specific phenome-wide association scan, the allele score of *CDKN1A* locus was significantly associated with HCM (odds ratio=1.82 [95% CI, 1.44–2.31] and Bonferroni-corrected *P*=9.83×10^−4^ per 1 SD increase in genetic risk score [GRS]). The allele scores of *FNDC3B* and *LINC02398* were significantly associated with cardiovascular risk factors such as hypercholesterolaemia and hypertension. *SYNPO2L* and *PXN* loci were associated with atrial fibrillation and flutter. The highly pleiotropic *MAP3K14* locus showed associations with diverse traits such as osteoarthritis, breast cancer, and hypothyroidism (Table S10).

### Association Between LVMWT GRS and Risk of HCM

**Figure 3.**
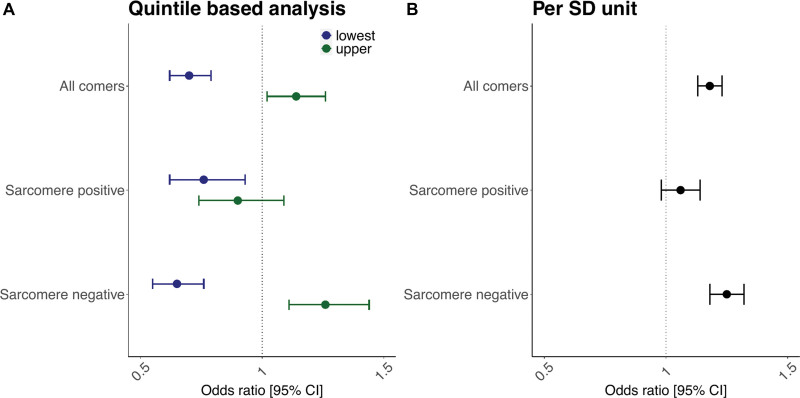
**Relationship between left ventricular maximum wall thickness (LVMWT) genetic risk score (GRS) and hypertrophic cardiomyopathy (HCM) status.** The GRS was evaluated in all-comers, sarcomere-positive and sarcomere-negative HCM cases. **A**, A quintile-based analysis demonstrates how a GRS of susceptibility loci underpinning LVMWT show protective effects in the lowest 20% of the population compared with the middle 60%. Similarly, the upper 20% show increased susceptibility towards a risk of developing HCM compared with the middle 60%. These associations appear driven in the all comer HCM analysis by sarcomere negative HCM. **B**, A per-SD estimate is reported to facilitate comparison with other GRSs. Odds ratios (*x* axis) are reported, with error bars denoting 95% CI.

**Figure 4.**
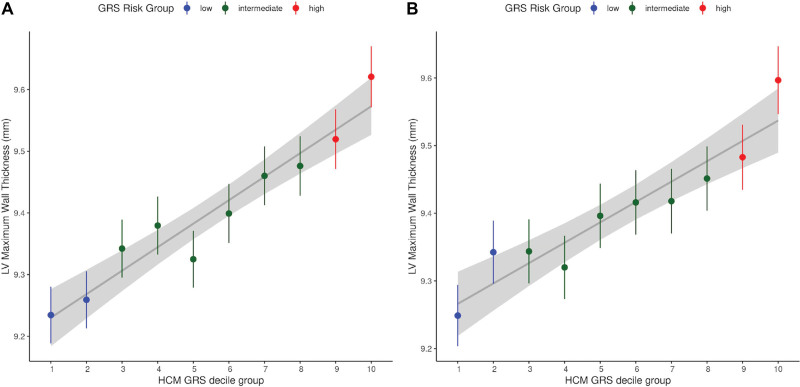
**Relationship between hypertrophic cardiomyopathy (HCM) genetic risk score (GRS) and phenotypic left ventricular maximum wall thickness (LVMWT). A**, The association between the HCM GRS from Tadros et al and LVMWT. **B**, The association between the HCMR GRS from Harper et al and LVMWT. The gray line with the shaded area indicates the linear regression estimate with its 95% CI. The dots and the vertical error bars represent the mean and 95% CI of LVMWT in each HCM GRS decile group. GRS indicates genetic risk score; HCM, hypertrophic cardiomyopathy; and LVMWT, left ventricular maximum wall thickness.

Given the role of LVMWT in HCM diagnosis and prognosis, we investigated the combined effect of susceptibility loci influencing LVMWT (Table S11) on the HCM case-control status in the Hypertrophic Cardiomyopathy Registry cohort. The LVMWT GRS contributed towards the risk of HCM (per SD odds ratio: 1.18 [95% CI, 1.13–1.23]; *P*=2.63×10^−13^; Table 2, Figure 3). This association appeared driven by sarcomere negative HCM (per SD odds ratio: 1.25 [1.18–1.32]; *P*=1.36×10^−15^), rather than sarcomere positive HCM (per SD odds ratio: 1.06 [0.98–1.14]; *P*=0.15). Conversely, there was a small but significant positive association between HCM GRS constructed from the SNPs reported by another HCM case-control study by Tadros et al^23^ (Table S12) and LVMWT in our cohort (per SD beta: 0.11 [0.09–0.12] mm; *P*=5.78×10^−63^). A directionally concordant association was observed in the sensitivity analysis using the HCM GRS from the Hypertrophic Cardiomyopathy Registry cohort (Table S13; per SD beta: 0.08 [0.07–0.09] mm; *P*=5.72×10^−39^; Figure 4).

**Table 2.**
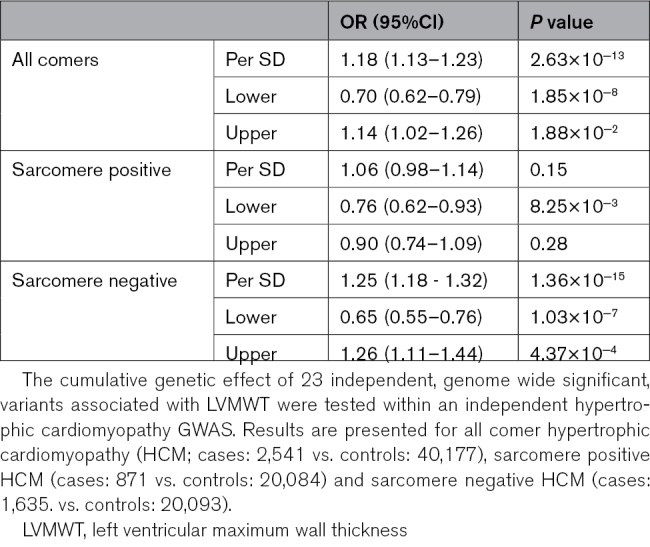
LVMWT Genetic Risk Score Tested Within Hypertrophic Cardiomyopathy

### Genetic Relationships Between LVMWT, SBP, and HCM Status

As LVMWT is a marker of LVH influenced by hypertension, we examined the extent of genetic similarities between LVMWT and systolic blood pressure (SBP) by LD score regression and GWAS-pairwise analyses. A modest genetic correlation exists between SBP and LVMWT (r_g_ [SE]=0.19 [0.03], *P*=6.0×10^−12^). Pairwise comparison of SBP and LVWMT GWAS summary statistics revealed 4 loci (*PTK2*, *SYNPO2L*, *HLF*, *MAPRE2*) harboring the same causal variant (posterior probability of association [PPA]_3>0.5). In 16 loci, different causal variants were identified for SBP and LVMWT (PPA_4>0.5; Table 1; Table S14).

A significant positive correlation between LVWMT and HCM case-control status (r_g_ [SE]=0.53 [0.09]; *P*=1.86×10^−8^) was observed using LD score regression. In the pairwise analysis, 18 out of 21 LVMWT loci harboured the same causal variants (PPA_3>0.5). One locus (*RAPSN*) showed a different causal variant (PPA_4>0.5); however, the repeat analysis using the summary statistics from HCM sarcomere-negative subset identified the same causal variant (PPA_3>0.5) for this locus (Table 1; Table S15).

## Discussion

This large-scale GWAS of LVMWT robustly measured from highly standardized CMR studies in 42 176 European individuals found SNP heritability of 26% and identified 21 genetic susceptibility loci. Variants in several LVMWT loci have known genetic associations with HCM and hypertension—an observation in part supported by the phenome-wide association scan—and other LV imaging measurements. Candidate genes for LVMWT loci are enriched in the pathways for sarcomere organisation and muscle development. Individuals with a LVMWT GRS in the top quintile of the population have increased odds of HCM relative to the middle 60% of the population. In the reverse direction, the HCM GRSs from two independent studies show a linear association with LVMWT. Pairwise comparison of GWAS data revealed a strong genetic relationship between LVMWT and HCM, where 85% to 90% of discovered loci carry the same causal variants. A more modest genetic correlation exists between LVMWT and SBP, which is underlined by a limited number (19%) of loci harbouring the same causal variants.

LVMWT is an important clinical phenotype widely used to quantify LVH and as a diagnostic and prognostic marker of HCM. Prior to this study, little was known about the genetic architecture of this important endophenotype. The genotype-attributable population variance of LVMWT (i.e. SNP heritability) is comparable to other LV phenotypes such as LV volume and LV ejection fraction which were previously reported to have 22% to 39% heritability.^19^ Variants in several LVMWT loci have known associations with related cardiac imaging measurements, ECG traits and cardiovascular diseases. These include *LINC00964* (in close proximity to *MTSS1*), *PXN*, *ALPK3*, and *MAP3K14* in association with LV volume, LV ejection fraction and LV mass; *CDKN1A*, *PTK2*, and *MAPRE2* in association with ECG traits (PR interval, QRS duration) and *SYNPO2L*, *LINC02398*, and *XPO7* in association with atrial fibrillation and coronary artery disease. To some extent, these associations are anticipated given the close physiological relationship between LVMWT and cardiac electrical and imaging traits and its known role, partly by reflecting abnormal cardiac remodelling, in predicting adverse cardiovascular outcomes.^2,27^ Several LVMWT loci (*SYNPO2L*, *PTK2*, *RAPSN*, *LINC02398*, *MAP2K14*) had prior associations with blood pressure traits in the published GWAS literature. It is possible that the observed genetic association between these loci and LVMWT is mediated by hypertension. However, further evaluation in the pairwise analysis revealed shared causal variants only in two loci (*SYNPO2L* and *PTK2*), suggesting distinct genetic underpinnings in the remaining overlapping loci.

Our investigation into the genetic relationship between LVMWT and HCM status identified a moderate genetic correlation (r_g_=0.53) and a significant genetic overlap where 18 out of 21 LVMWT loci shared the same causal variants. This finding supports the strategy of using the common-variant analysis in a related endophenotype to help uncover the etiopathology of HCM which remains incompletely understood. Locus-trait associations observed in an all-comer HCM population may be influenced by synthetic association with underlying rare causal variants.^28^ Here, the *RAPSN* locus, which is located in close proximity (i.e. within the range of an LD block [r^2^>0.5]) to *MYBPC3*, which is the most frequent causal gene for HCM,^29^ was deemed to possess two differing causal variants when LVMWT and all-comer HCM were assessed. When only sarcomere-negative HCM and LVMWT were analyzed, a single sentinel variant emerged within the *RAPSN* locus.

### Candidate Genes

Based on bioinformatics analysis, available animal models, functional studies, and published literature, we have found several promising candidate genes amongst 80 candidate genes in 21 loci. Beyond the recognized causal genes for Mendelian cardiomyopathy such as *FHOD3*^30^ and *ALPK3*,^31,32^ a number of prioritised genes merit further discussion. *PROX1* (Prospero Homeobox 1) gene in the novel *PROX1* locus, was previously reported as an important regulator of cardiac development and myocardial growth.^33,34^ A knock-out mouse model (Mouse Genome Informatics:97772) showed myofibrillar disarray, chamber dilatation and systolic dysfunction, which are all relevant cardiomyopathy-related traits. *PXN* (Paxillin) gene in the *PXN* locus is a cytoskeletal protein implicated in actin-membrane attachment at sites of cell adhesion to the extracellular matrix. This gene was reported to be involved in pathways of sarcomere organisation and cardiomyocyte hypertrophy^35^ with a mouse model (Mouse Genome Informatics:108295) showing abnormal heart development. *PTK2* (Protein Tyrosine Kinase 2) gene in the *PTK2* locus has a role in cell migration, organization of the cytoskeleton, and is needed for embryonic angiogenesis, normal cardiomyocyte migration and proliferation, and normal heart development. Functional work involving this gene demonstrated its function in cardiac myocyte hypertrophy in response to stress.^36–40^ Its biological relevance was further reinforced in a mouse model (Mouse Genome Informatics:95481) showing abnormal myocardial fiber morphology, disarray, hypertrophy and interstitial fibrosis. Overall, these candidate genes are involved in sarcomere/myofibril development and maintenance, cardiac developmental pathways and/or cardiomyocyte hypertrophy as additionally supported by the g:Profiler pathway analysis.

### The Genetic Risk Score for LVMWT Predicts the Risk of HCM

Although commonly considered a Mendelian disorder with autosomal dominant transmission, family studies in individuals carrying sarcomere mutations often show incomplete penetrance and variable expressivity. Also, many cases appear to be sporadic and even in the presence of family history, the yield of genetic testing for the established causal sarcomere genes is ~50%.^41^ Family-based linkage analyses and more recently whole-exome sequencing projects have mostly failed to discover new causal genes. These data are indicative of a more complex inheritance model in a proportion of mutation-negative patients. Common variants acting through a polygenic effect contribute to the phenotype of other inherited conditions including familial hypercholesterolaemia^42^ and Brugada syndrome.^43^ One of the few such studies focusing on common variations in HCM^44^ led to the discovery of *FHOD3*^30^ recently described as a Mendelian cause of the disease. *FHOD3* was also highlighted as a candidate for LVMWT in this study.

Two other GWASs in HCM cohorts have been published recently. In the first study,^22^ a GRS derived from an HCM case-controls analysis was shown to be associated with the risk of developing HCM in three validation cohorts. This risk was more marked for sarcomere-negatives than positives, although significant in both sub-cohorts. In our current work, a GRS derived from 23 SNPs associated with LVMWT showed a similar association with the risk of HCM in the Hypertrophic Cardiomyopathy Registry population. The association was evidently stronger in the sarcomere-negative HCM, although it should be noted that the statistical power was more limited in the sarcomere-positive group due to a smaller sample size. This reflects the fact that LVMWT is the sole morphological diagnostic criterion and defining trait of HCM. It also further confirms the relevance of an oligogenic/polygenic model especially in the pathogenesis of sarcomere-negative HCM, where the effects of higher penetrance Mendelian mutations are absent in the majority of patients, who tend to be older, have lower prevalence of a family history and in general a more benign phenotype^45^ compared to the sarcomere-positives. Looking at the opposite direction, a positive association was identified between the GRS derived from HCM case-control analysis and phenotypic LVMWT in the UK Biobank cohort, again reflecting the overlap between the genetic architecture of HCM and LVMWT. This bidirectional relationship was further underlined by a significant and positive genotypic correlation and a large proportion of shared causal variants in the pairwise loci lookup.

It is perhaps surprising to find a broad genetic overlap between the LV wall thickness measured in a mostly healthy community-dwelling population and HCM based on the data from HCM registries, which, by definition, included individuals with extreme wall thickness. One could postulate that the genetic associations captured in the HCM case-control studies are mostly reflecting the cumulative genetic burden of LVMWT as a physiologic trait. Additionally, some of these loci could possibly reflect an association with hypertension and its effect on LV wall thickness.

### Strengths and Weaknesses

A key strength of our work is the availability of highly precise CMR-derived LV wall thickness measurements enabled by a robust deep learning algorithm coupled with an additional layer of image quality review by experienced Cardiologists. This permitted a well-powered genome-wide analysis which unraveled previously unknown genetic elements influencing the individual variation in LVMWT. By leveraging the summary data from large blood pressure and HCM GWASs, we were able to illuminate the genetic relationships between LVMWT and SBP and HCM risk. However, a few limitations of this study should be acknowledged. To maximize the power of discovery, we used a single-stage study design. The reported loci should be replicated in future independent cohorts to lend further credibility to our results. Furthermore, as our analysis was largely of European ancestry, caution should be taken when interpreting the relevance of our findings in non-European populations.

### Future Directions

The novel candidate genes and related pathways could indicate new candidate genes for monogenic HCM. Rare variants in these genes should be tested in large cohorts of cardiomyopathy patients for its presence, segregation in families and enrichment versus controls. Additionally, the newly identified proteins encoded by the candidate genes could be used in druggable target evaluation approaches (for example by employing Mendelian randomisation methodology), to help develop new disease-modifying therapies. A polygenic risk score developed from a meta-analysis of the different studies could enter clinical practice and be used as part of the genetic testing and screening of HCM patients and relatives to assess the likelihood of developing this condition—this might be particularly significant in sarcomere-negatives but also in sarcomere-positives carrying the same main causative variant, where the GRS would act with a modifier effect. Future work could also evaluate if a consensus GRS is predictive of adverse outcomes including heart failure and arrhythmia in HCM patients, in integration with rare variation. It remains to be explored whether the genetic predisposition to increased LVMWT is associated with other pathological contexts such as hypertensive heart disease and aortic stenosis, where LVWMT as a surrogate marker for LVH is known to have prognostic relevance.^46,47^ Last, studies involving multi-ancestral populations are needed to understand the robustness and generalisability of our findings in non-European ethnicities.

In summary, this genome-wide association study of LVMWT discovered 21 genomic loci enriched with genes involved in sarcomere organisation and myocyte development and highlights the shared genetic background with HCM. These findings provide new avenues for future research endeavours to elucidate the genetic determinants of HCM.

## Article Information

### Acknowledgments

This study was conducted using the UK Biobank resource under access application 2964. We would like to thank all the participants, staff involved with planning, collection and analysis, including core lab analysis of the CMR imaging data. Dr Aung contributed to the conceptualization, data curation, formal analysis, investigation, methodology, resources, software, validation, visualization, writing, reviewing, and editing the original draft. Dr Lopes contributed to the conceptualization, investigation, methodology, writing, reviewing, and editing the draft. Dr van Duijvenboden contributed to data analysis and article drafting. Drs Harper and Goel contributed to data curation and analysis, validation, reviewing, and editing the draft. Dr Grace, Ho, Weintraub, Kramer, and Neubauer contributed in the methodology, resources, reviewing, and editing the draft. Drs Watkins, Petersen, and Munroe contributed to the conceptualization, funding acquisition, methodology, project administration, supervision, reviewing, and editing the original draft. Data and code availability: The LV image-derived data and other results generated in this study will be available from the UK Biobank data repository, which can be accessed by researchers upon application. The GWAS summary data will be available in GWAS Catalog after publication.

### Sources of Funding

Dr Aung recognizes the National Institute for Health and Care Research (NIHR) Integrated Academic Training programme, which supports his Academic Clinical Lectureship post and acknowledges the support from an Academy of Medical Sciences Starter Grant for Clinical Lecturers, which enabled the computational experiments. Dr Lopes is funded by a Medical Research Council (MRC) Clinical Academic Research Partnership (CARP) award (MR/T005181/1). Dr Kramer acknowledges funding from National Heart, Lung, and Blood Institute (NHLBI) grant U01 HL117006-01A1. Dr Grace is funded by British Heart Foundation (BHF) chair award (HSR01000). His research is supported by the Wellcome Trust Core Award Grant Number 203141/Z/16/Z with additional support from the NIHR Oxford Biomedical Research Centre (BRC). Dr Neubauer acknowledges support from the National Heart, Lung, and Blood Institute grant U01HL117006-01A1. Drs Neubauer and Watkins acknowledge support from the Oxford NIHR Biomedical Research Centre and the Oxford BHF Centre of Research Excellence. Dr Goel acknowledges funding from the Wellcome Trust core award (090532/Z/09/Z, 201543/B/16/Z); HEALTH-F2-2013-601456 (CVGenes@Target), the TriPartite Immunometabolism Consortium [TrIC]-Novo Nordisk Foundation’s Grant number NNF15CC0018486, VIAgenomics (SP/19/2/344612). A.H. was supported by the MRC Doctoral Training Partnership when the underlying data were generated. Drs Petersen and Munroe acknowledge support from the NIHR Biomedical Research Centre at Barts. Dr Petersen has received funding from the European Union’s Horizon 2020 research and innovation programme under grant agreement No. 825903 (euCanSHare project). Dr Petersen also acknowledges support from and from the “SmartHeart” Engineering and Physical Sciences Research Council (EPSRC) programme grant (www.nihr.ac.uk; EP/P001009/1). Dr van Duijvenboden acknowledges funding support from the Queen Mary University of London (QMUL) Impact Acceleration Accounts. Drs Petersen and Neubauer acknowledge the BHF for funding the manual analysis to create a cardiovascular magnetic resonance imaging reference standard for the UK Biobank imaging resource in 5000 CMR scans (www.bhf.org.uk; PG/14/89/31194).

### Disclosures

Dr Kramer reports consulting fees from Bristol Myers Squibb and Cytokinetics outside of the submitted work. Dr Harper is an employee of Astra Zeneca and owns stock option in Astra Zeneca. Dr Lopes reports consulting fees from Bristol Myers Squibb outside of the submitted work.

### Supplemental Material

Supplemental Methods

Tables S1—S15

Figures S1—S5

References^48^–^69^

## Supplementary Material


