# Supplementary material for: Genome-Wide Analysis of Left Ventricular Maximum Wall Thickness in the UK Biobank Cohort Reveals a Shared Genetic Background With Hypertrophic Cardiomyopathy
Source: Circ Genom Precis Med. 2023 Jan 4;16(1):e003716. doi: 10.1161/CIRCGEN.122.003716 (PMC9946169; doi:10.1161/CIRCGEN.122.003716)
Supplement: Supplementary file 1 [file hcg-16-e003716-s001.pdf]

SUPPLEMENTAL MATERIAL

Supplemental Methods

UK Biobank

The UK Biobank (UKB) is a prospective study of ~500,000 volunteers from a general population, comprising men and women aged 40 to 69 years at the time of initial recruitment between 2006 and 2010. Extensive baseline and follow-up clinical, biochemical, genetic and outcome measures are available. Genotyping was performed by UKB using the Applied Biosystems (Affymetrix) UK BiLEVE Axiom Array or the UKB Axiom™ Array. Genotype imputation was performed centrally using the Haplotype Reference Consortium and the merged UK10K and 1000 Genomes phase 3 (1000G) reference panels. The study protocol has been described in detail previously[25]. In this study, we included the sub cohort of 46,315 individuals with available cardiovascular magnetic resonance imaging (CMR) data from the ongoing UKB imaging sub-study.

Derivation of maximum wall thickness from CMR

Detailed CMR protocol and analysis methods have been described previously[50,51]. We adapted a deep convolutional neural network[18] developed from 4,875 manually annotated UKB CMR studies to automatically segment the LV epicardium and endocardium in the short axis steady-state free precession (SSFP) cine images acquired with wide-bore 1.5 Tesla scanners (MAGNETOM Aera, Siemens Healthcare, Erlangen, Germany). The maximum distance between LV epicardial and endocardial contours is calculated in each of the 16 segments from the American Heart Association (AHA) model in the end-diastolic phase. We recorded the maximum value of these 16 measurements as the LVMWT (Figure 1).

UK Biobank sample selection

We excluded CMR studies with incomplete LV coverage (N slices < 6) and inadequate segmentation quality (number of segmented voxels < 150 for the basal slices and < 50 for the apical slices). Additionally, all CMR studies with: (i) outlying values defined as 3 x interquartile range above and below the first and third quartile, respectively for unindexed and body-surface-area and height indexed LV volumes, LV mass and LVMWT and (ii) non-physiological measurements (LV end-diastolic volume < 75ml, LV end-systolic volume < 25ml and LV mass < 40g) and (iii) LVMWT ≥ 13mm were visually reviewed by two European Association of Cardiovascular Imaging (EACVI) level-3 certified analysts (N.A. and L.R.L.) to ascertain the accuracy of measurements.

We next applied a series of genotypic quality control checks (QC) to individuals with good quality CMR studies as outlined in Supplemental Figure 4. We removed individuals with missing genotype (N = 1,066), discordance between the self-reported and genetically inferred sex (N = 26), poor genotype quality (N = 79) and non-European ancestry (N = 1,492).

Genetic analyses

We first selected a set of high quality directly genotyped variants known as model single nucleotide polymorphisms (model SNPs) by applying the following filters: a minor allele frequency (MAF) > 5%, a Hardy–Weinberg equilibrium (HWE) threshold of P-value $=1\times 10^{-6}$, and missingness < 0.0015. The LVMWT phenotype was normalised using rank-

based inverse normal transformation as it showed evidence of positive skewness (Supplemental Figure 5). We estimated the heritability explained by genotype (h_g^2 SNP) using BOLT-REML software [52]. We next performed the discovery GWAS in a linear mixed-model method by BOLT-LMM software [53] using the model SNPs (329,861 variants) and ~ 9.9 million imputed variants with MAF ≥ 1% and INFO > 0.3. Both heritability and GWAS models were adjusted for age, sex, body surface area, mean arterial blood pressure corrected for antihypertensive medication use (by adding 15 mmHg to systolic blood pressure and 10mmgHg to diastolic pressure), genotyping array type (UK Biobank versus UK BiLEVE array), and imaging centre. A GWAS P value threshold of $< 5 \times 10^{-8}$ represents genome-wide significance. We defined a genomic locus as a region encompassing 500kb upstream and downstream of the lead variant with the smallest P value.

Conditional analysis

We examined the existence of secondary independent variants tagging the same GWAS loci by performing conditional analysis in genome-wide complex trait analysis (GCTA) software[54]. A secondary signal was declared if: (i) the newly identified variant's original GWAS P value was lower than 1×10^{-6}; (ii) there was < 1.5-fold difference between the lead variant and secondary association P values on a -log10 scale (i.e., if $-\log10(P_{lead})/-\log10(P_{sec}) < 1.5$); and (iii) if there was < 1.5-fold difference between the main association and conditional association P values on a -log10 scale (i.e., if $-\log10(P_{sec})/-\log10(P_{cond}) < 1.5$).

Percent variance

The proportion of variance explained by the genome-wide significant WT loci was calculated by the difference in adjusted R^2 between the linear regression model containing all covariates plus all lead variants and the model containing only analysis covariates.

Variant annotation

99% credible sets were created for each genome-wide significant locus using the Bayesian approach previously described by Wakefield[55]. We used Variant Effect Predictor (VEP) tool (v103)[56] to describe the type, consequence and predicted function based on SIFT and PolyPhen-2 of all variants in the 99% credible sets. Non-synonymous variants were considered 'damaging' if concordantly predicted as having detrimental effects by both SIFT and PolyPhen-2. For non-coding variants, CADD (v1.6)[57] and RegulomeDB (v2.0)[58] databases were used to ascertain functional relevance. Variants with scaled CADD score > 20 or RegulomeDB score ≤ 3 were considered functionally important. Fine-mapping of causal variants within credible sets was performed by the colocalisation analysis of GWAS and cis-eQTL signals from cardiovascular tissues (aorta, coronary artery, left atrial appendage and left ventricle) in GTEx (v8) [59] using coloc R package [60].

Pleiotropy analyses

We queried the lead variants and their close proxies (linkage disequilibrium [LD] $r^2 \geq 0.8$) against PhenoScanner (v2)[61] and GWAS Catalog (queried on 22th August 2021)[62] to investigate trait pleiotropy. We also investigated the overlap of loci discovered in our LVMWT GWAS and a recently published LV mean wall thickness GWAS[24] in the same UKB cohort.

Gene prioritisation

Long-range chromatin interaction (Hi-C) analysis

To identify distal candidate genes, we explored chromatin interaction (promotor capture Hi-C) data[63] for all lead and secondary variants and their proxies with regulatory potential (RegulomeDB score ≤ 3). We identified several genes whose promoter regions form significant chromatin interactions in heart atria, ventricles, and aorta.

Candidate genes at each locus were then collated based on evidence from:

1. presence of damaging coding variant at the locus;
2. genes prioritised by the eQTL colocalisation analysis;
3. targets genes from Hi-C data;
4. genes prioritised by DEPICT[64] (Data-driven Expression-Prioritised Integration for Complex Traits);
5. availability of knockout model from International Mouse Phenotyping Consortium (http://www.mousephenotype.org/) and the Mouse Genome Informatics database (http://www.informatics.jax.org/) with a cardiovascular phenotype;
6. genes which are nearest to or locating within the 10kb window of the lead variant or the region of LD block $r^2 > 0.5$, using University of California, Santa Cruz (UCSC) known genes database

Gene-set and pathway enrichment analyses

We performed an unbiased gene-set and pathway enrichment analysis of GWAS signals in DEPICT using the LVMWT GWAS summary statistics with an association P value threshold of 1 x 10^{-5} as recommended by the authors of DEPICT. Additionally, we used the g:Profiler tool[65] to investigate if our prioritised genes (supported by at least 2 lines of evidence as described above) are over-represented in a particular biological pathway. In g:profiler, multiple-testing correction was performed by the bespoke ontology-focused g:SCS (Set Counts and Sizes) method[66] at 5% threshold.

Phenome-wide association study

We examined the associations between the variants for LVMWT and other phenotypes by conducting a Phenome-wide association study (PheWAS) in unrelated individuals of European ancestry from UK Biobank (N = 343,849) not included in our LVMWT GWAS. Logistic regression analyses adjusted for age, sex and the first ten genetic principal components (PCs) were performed for each locus-specific weighted allele scores and 1,513 outcome phenotypes based on the hospital episode statistics defined according to the phecode system as previously described[67]. Bonferroni correction was applied to the PheWAS P values (adjusted $P < 3.3x10^{-5}$).

HCMR

HCMR is a registry of 2,755 incident HCM cases, aged 18 to 65 years old with evidence of unexplained left ventricular hypertrophy (wall thickness >15mm), recruited from 44 sites across six countries in North America and Europe[68]. As described in Harper et al.[23], individual-level genotype data, imputed using the Haplotype Reference Consortium panel,

were available for 2,541 HCMR cases of European ancestry. Controls (n=40,177) were randomly sampled from distantly related UKB participants, using 20:1 allocation, following exclusion of those participating in the UKB CMR sub-study, and with approximate age, sex and ancestry matching[23]. HCMR cases were dichotomized based on the presence of a causal genetic variant in a core sarcomere gene (*ACTC1*, *MYBPC3*, *MYH7*, *MYL2*, *MYL3*, *TNNT2*, *TNNI3* and *TPM1)* into sarcomere positive (n = 871) and sarcomere negative (n = 1,635) groups[68].

Association between LVMWT GRS and HCM

A genetic risk score (GRS) that combined independent (i.e. in linkage equilibrium) effects of primary and secondary variants achieving genome wide significance in the LVMWT analysis were tested in an independent, European, HCM GWAS (HCM cases: 2,541; UKB controls: 40,177)[23]. Participants undergoing CMR imaging within UKB were not included in the HCM GWAS analysis. The cumulative genetic effect of the 23 variants (h^2=1.17%; F statistic = 21.7) was calculated for each individual using the --risk-score function in QCTOOL v2. The relative weight assigned to each variant was the β estimate from the UKB European LVMWT GWAS. A logistic regression model was fitted with affection status as the outcome variable and standardised GRS score as an explanatory variable, with covariates including the first ten genetic PCs, age and sex. Cases from the HCMR dataset were dichotomised based on the presence of a rare causal variant in an established sarcomere gene into sarcomere-positive and sarcomere-negative cases, as previously reported[69]. Per standard deviation and quintile-based analyses were performed to assess the impact.

We also investigated the association between the HCM GRS and phenotypic LVMWT using the effect sizes of genome-wide significant SNPs from a large HCM GWAS meta-analysis by Tadros et al[24]. We fitted a logistic regression model adjusted for age, sex, and the first ten genetic PCs as covariates. In a sensitivity analysis, we repeated the regression model using the HCM GRS constructed from the weights of SNPs with false discovery rate (FDR) < 0.05 in Harper et al.[23] (the HCMR case-control GWAS).

Genetic correlation and pairwise comparison of LVMWT, systolic blood pressure and HCM

LD score regression (LDSC)[70] was applied to estimate the pairwise genetic correlations using the summary association statistics of LVMWT, systolic blood pressure[71] (SBP) and HCM[23]. SBP summary data was obtained from a meta-analysis comprising 757,601 European individuals from the International Consortium of Blood Pressure (ICBP) and UK Biobank[71]. GWAS-PW[72] software was used to screen the GWAS summary statistics for loci driven by either: the same causal variant (model 3), different causal variants (model 4), only one GWAS shows association (model 1 or 2), or unresolvable (no SNPs in the region that influence either trait). The software uses a Bayesian approach to estimate log likelihood for a given region for each of the four models. We inferred the presence (model 3) or absence (model 4) of shared genetic effects based on a posterior probability threshold greater than 0.5 (50%) in the two models for each of the 21 LVMWT loci.

Supplemental Tables (see separate Excel File)

Supplemental Table I. Cohort characteristics of the UK Biobank
Supplemental Table II. Conditional analysis in GCTA
Supplemental Table III. Summary of variant-level annotations
Supplemental Table IV. Lookup of the WT GWAS lead variants and their proxies (LD ≥ 0.8) in Phenoscanner v2
Supplemental Table V. Lookup of the WT GWAS lead variants and their proxies (LD ≥ 0.8) in GWAS Catalog
Supplemental Table VI. Lookup of the WT GWAS lead variants and their proxies (LD ≥ 0.8) in the GWAS summary statistics for mean wall thickness with P<1x10-5 (Tadros et al. 2020)
Supplemental Table VII. Summary of candidate genes
Supplemental Table VIII. DEPICT gene set enrichment analysis based on all independent SNPs with P value < 1e-5
Supplemental Table IX. Significant gene sets and pathways in g:Profiler
Supplemental Table X. Phenome-wide association studies of WT loci
Supplemental Table XI: Independent variants used for LV maximum wall thickness genetic risk score
Supplemental Table XII: Independent variants used for HCM genetic risk score from Tadros et al.
Supplemental Table XIII: Independent variants used for HCM genetic risk score from Harper et al.
Supplemental Table XIV. GWAS-PW results for LVMWT and SBP summary statistics
Supplemental Table XV: GWAS-PW results for LVMWT and HCM summary statistics

Supplemental Figures

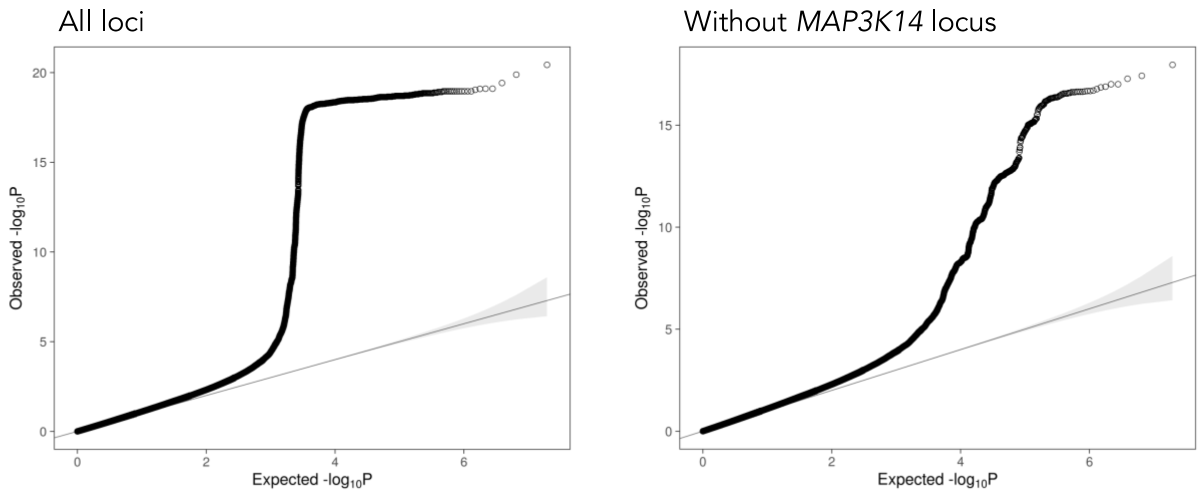

Supplemental Figure I. Quantile-quantile plots of LV maximum wall thickness GWAS P values for all loci and without *MAP3K14* locus

Unadjusted P values are two-sided based on the Chi-square test statistics in the BOLT-LMM software.

LV, left ventricle; GWAS, genome-wide association study; λ, genomic inflation factor

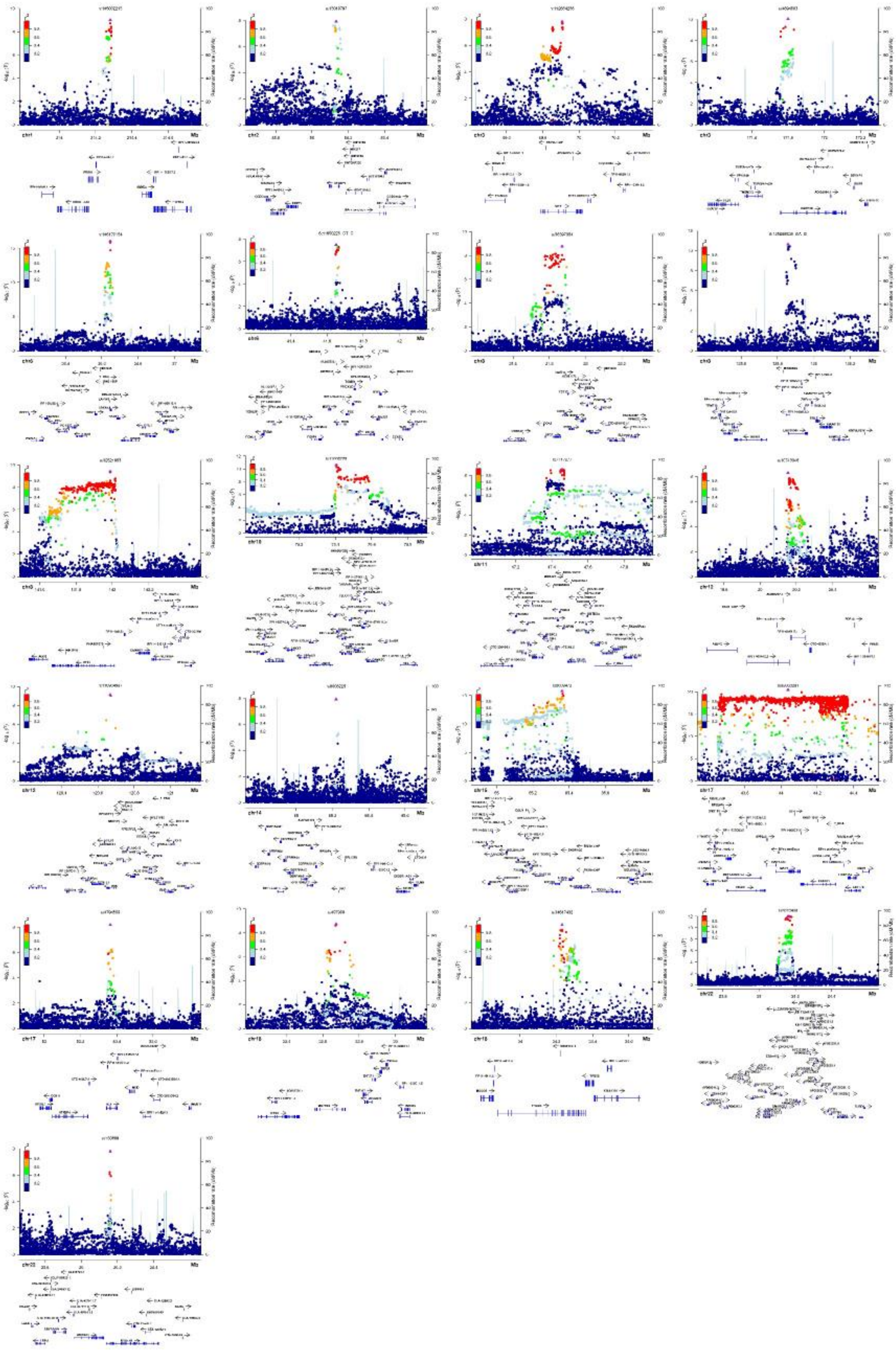

Supplemental Figure II. LocusZoom plots of LV maximum wall thickness GWAS loci

Each point represents a genetic variant. The LocusZoom regional plot illustrates the GWAS association P values on the -log10 scale on the left-hand vertical axis, the recombination rate on the right-hand vertical axis, and the chromosomal position along the horizontal axis.

LV, left ventricle; GWAS, genome-wide association study

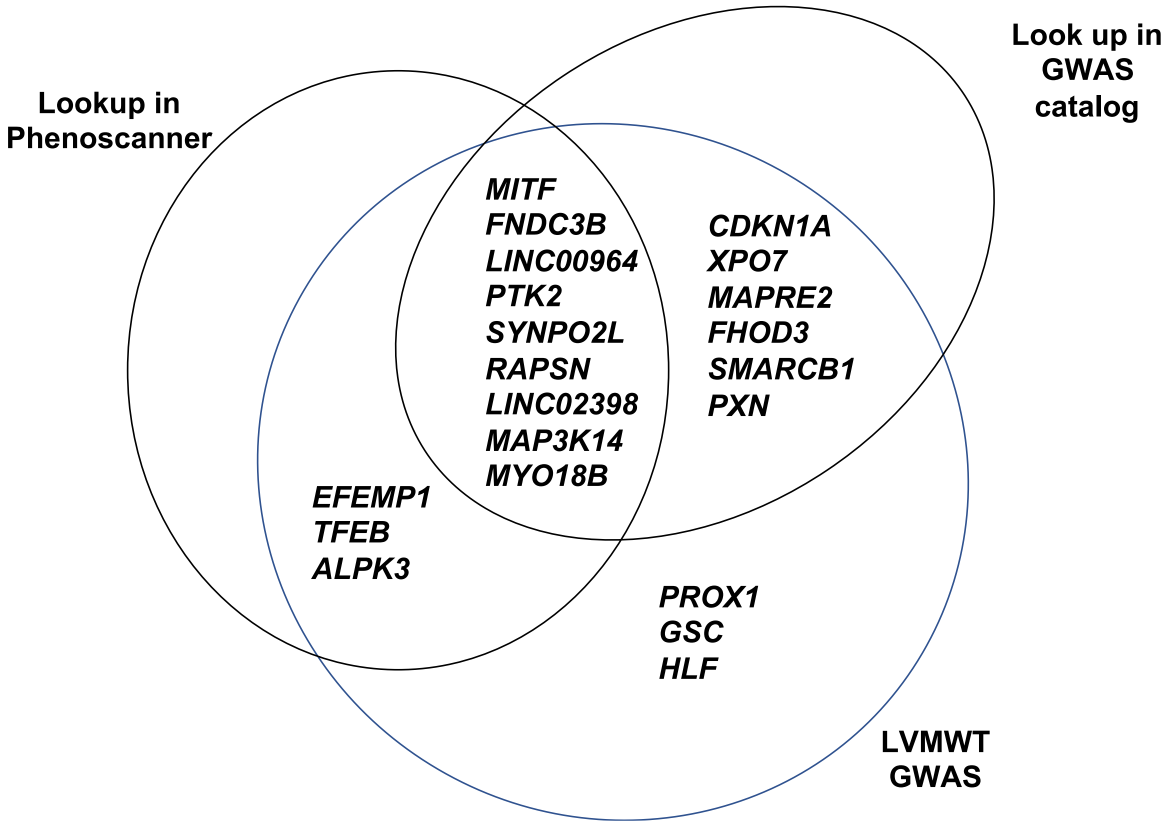

Supplemental Figure III. Overlap of LVMWT loci with previously reported associations from Phenoscanner and GWAS Catalog

Venn diagram demonstrates the overlap of LVMWT loci with the loci reported in association with other traits. The locus name indicates the nearest annotated gene.

LVMWT, left ventricular maximum wall thickness

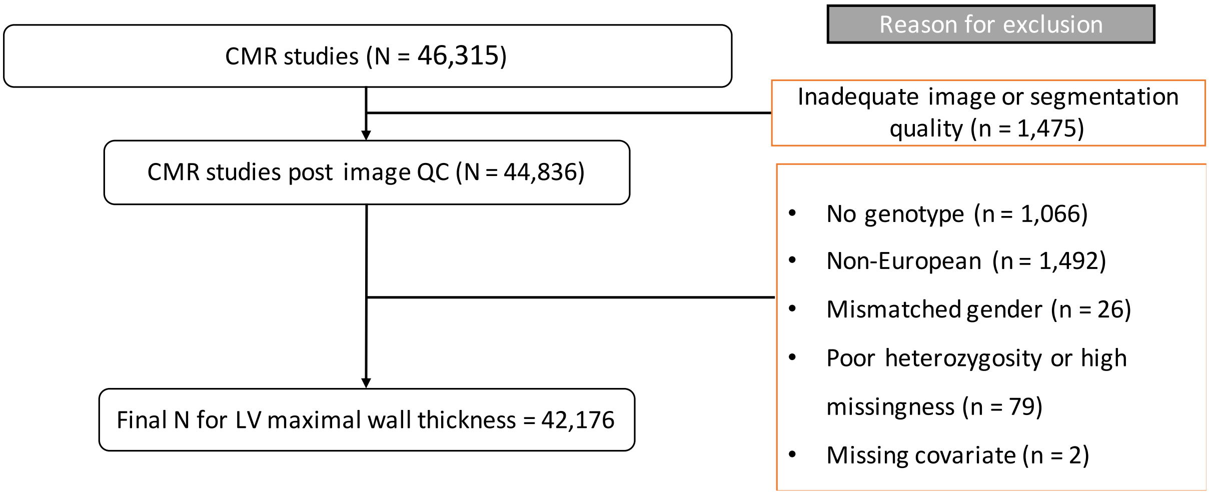

Supplemental Figure IV. Sample selection flowchart

CMR, cardiovascular magnetic resonance; QC, quality control; LV, left ventricle

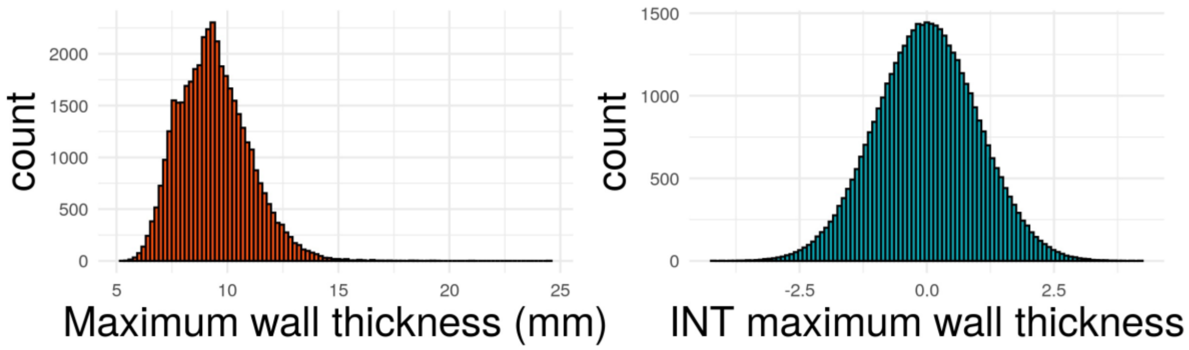

Supplemental Figure V. Distribution of untransformed and transformed LV maximum wall thickness

Histograms of untransformed and INT LV maximum wall thickness.

INT, rank-based inverse normal transformation; LV, left ventricle
